# Assessing agreement between different polygenic risk scores in the UK Biobank

**DOI:** 10.1038/s41598-022-17012-6

**Published:** 2022-07-27

**Authors:** Lei Clifton, Jennifer A. Collister, Xiaonan Liu, Thomas J. Littlejohns, David J. Hunter

**Affiliations:** 1 Nuffield Department of Population Health, University of Oxford, Oxford, UK; 2 Department of Epidemiology, Harvard TH Chan School of Public Health, Boston, MA USA

**Keywords:** Epidemiology, Genetics research, Translational research

## Abstract

Polygenic risk scores (PRS) are proposed for use in clinical and research settings for risk stratification. However, there are limited investigations on how different PRS diverge from each other in risk prediction of individuals. We compared two recently published PRS for each of three conditions, breast cancer, hypertension and dementia, to assess the stability of using these algorithms for risk prediction in a single large population. We used imputed genotyping data from the UK Biobank prospective cohort, limited to the White British subset. We found that: (1) 20% or more of SNPs in the first PRS were not represented in the more recent PRS for all three diseases, by the same SNP or a surrogate with R^2^ > 0.8 by linkage disequilibrium (LD). (2) Although the difference in the area under the receiver operating characteristic curve (AUC) obtained using the two PRS is hardly appreciable for all three diseases, there were large differences in individual risk prediction between the two PRS. For instance, for each disease, of those classified in the top 5% of risk by the first PRS, over 60% were not so classified by the second PRS. We found substantial discordance between different PRS for the same disease, indicating that individuals could receive different medical advice depending on which PRS is used to assess their genetic susceptibility. It is desirable to resolve this uncertainty before using PRS for risk stratification in clinical settings.

## Introduction

Genome-wide association studies (GWAS) have revealed that the inherited genetic component of most traits not due to variations in a single gene is highly polygenic. Dozens or thousands of single nucleotide polymorphisms (SNPs) can be combined to produce a polygenic risk score (PRS) representing an individual's genetic propensity for a given trait or disease.

There is much enthusiasm for the use of PRS to inform individuals about their risk of future health conditions, either as stand-alone information, or combined with non-genetic data in integrated risk scores^1,2^. PRS have been proposed in a wide variety of settings such as the prioritisation of people for disease screening, informing the prescription of preventive medicines, and even in embryo selection^3^. Variants in single genes associated with diseases are already utilised in a clinical setting, however, recent large-scale studies have found that PRS could potentially identify a greater proportion of at risk individuals^4^.

Early in the development of PRS, researchers^5,6^ quantified the degree to which a PRS with a limited number of SNPs would misclassify people, when compared with future PRS with many additional variants. At the time it was thought that once dozens, or even hundreds of SNPs were included, diminishing returns would set in and the PRS would be relatively stable. This perception appeared to be supported by the fact that in many large multi-study consortia, additional SNPs now being identified have very small odds ratios (OR; as low as 1.02 per allele or less) and the area under the receiver operating characteristic curve (AUC) of newer versions of the PRS are only minimally higher than that of previous versions.

Recent discussion on the use of PRS in the clinic has largely focused on the reporting standards for the derivation and archiving of PRS^7,8^, the health economic value of PRS, the potential contribution of PRS to health disparities given the limited databases available for non-European ancestry populations^9^, how to use PRS for patient benefit^10,11^, and the means of communicating PRS to patients or members of the public.

However, in addition to the implementation of PRS in practice, it is important to consider the methodological choices made when deriving different PRS. During the construction of PRS, there are multiple design options for deciding the number of SNPs to include and for assigning an appropriate weight to each SNP. Consequently, multiple sets of SNPs exist, resulting in multiple PRS for the same trait. For example, the 313-SNP PRS for breast cancer^12^ was developed using hard-thresholding stepwise forward regression, whereas the 118 k-SNP PRS^13^ was selected by the penalised regression "lassosum" and the highest pseudo-R^2^. However, these PRS are typically compared at a population level using metrics such as the AUC or OR, and limited attention has been paid to how they differ from each other for risk prediction of individuals. Therefore, it is important to understand how the use of different PRS affect an individual’s classification of risk for future disease, as this has important implications for the use of PRS in wider practice.

## Materials and methods

### Study populations

We used the data from the UK Biobank (UKB), a large-scale population-based prospective cohort study of approximately 500,000 individuals aged 40–69 years at recruitment across the United Kingdom between March 2006 and October 2010. The full details of the genotyping and imputation are described elsewhere^14,15^.

Our study populations for each of the three disease outcomes are defined as follows:

- The breast cancer eligible population was women who had not had breast cancer, carcinoma in situ or mastectomy prior to baseline.
- For the hypertension eligible population, we excluded individuals with missing or implausible systolic blood pressure (SBP) measurements (< 70 or > 270 mmHg) at baseline, and those with Major Adverse Cardiovascular Events (MACE) prior to baseline.
- The dementia eligible population was restricted to individuals without a diagnosis of dementia prior to baseline.

We further restricted to genetically White British individuals (UKB Data Field 22006), and excluded individuals who were related (3rd degree or higher), sex discordant, or outliers for genotype missingness or heterozygosity based on UK Biobank-derived sample quality control data (UKB Data Field 22020). This yields the final size N of each study population.

Disease ascertainment in UKB during the follow-up period utilised linkage to death registry, cancer registry, and Hospital Episode Statistics (HES). Hypertension is defined as SBP >= 140 at baseline; the International Classification of Diseases (ICD) codes for breast cancer and dementia can be found in Supplementary Tables 14–16.

### Selection of PRS

For each of the three disease outcomes, we selected a pair of recently published PRS to compare, typically published within two years of each other. The earlier PRS is denoted as PRS-A, while the more recent one is PRS-B.

When choosing PRS we identified scores that were derived using the same trait definition in primarily White European populations, to be appropriate for use in the UK Biobank, and that had been derived in large consortia for their respective disease. Where possible, we selected scores listed in the Polygenic Score Catalog (PGS Catalog), an online database that collects and curates PRS from across the literature and makes metadata available in a standardised way.

Table 1 summarises the characteristics of the chosen PRS for each trait, including the construction method. Supplementary Table 1 contains further information about each PRS, including source of weights (i.e. derivation dataset), population characteristics, and validation dataset.

- For breast cancer, PRS-A (313 SNPs, PGS ID: PGS000004)^12^ has been widely validated and is included in the current implementation of the BOADICEA breast cancer risk model^16,17^. For PRS-B, we used a score (118,388 SNPs, PGS ID: PGS000511)^13^ which was largely developed from the same Breast Cancer Association Consortium (BCAC) GWAS data as PRS-A^18^.
- For hypertension, PRS-A for SBP (267 SNPs, not in PGS Catalog)^19^ was selected from the literature (further details later in this Section). The effect sizes of PRS-B for SBP (884 SNPs, PGS ID: PGS000812)^20^ are derived from the International Consortium of Blood Pressure-Genome Wide Association Studies (ICBP), Million Veteran Program (MVP) and Estonian Genomic Centre of the University of Tartu (EGCUT).
- For dementia, both PRS-A (57 SNPs, PGS ID: PGS000812)^21^ and PRS-B (39 SNPs, PGS ID: PGS001775)^22^ used effect sizes from the International Genomics of Alzheimer’s Project (IGAP) GWAS^23^. PRS-A was constructed in January 2021 while PRS-B was developed in September of the same year. We selected PRS that did not contain the two APOE SNPs (rs429358 and rs7412), in order to prevent the APOE genotype from dominating the PRS.

**Table 1:** PRS chosen for each condition.

Disease	PRS	nSNPs	Source (PGS ID)	Trait	Construction
Breast cancer	A	313	Mavaddat2019 (PGS000004)	Overall breast cancer	Hard-thresholding and stepwise forward regression, *p* < 10^−5^
	B	118,388	Fritsche2020 (PGS000511)	Overall breast cancer	Lassosum, s = 0.5, lambda = 0.004281
Hypertension	A	267	Warren2017 (Not in PGS Catalog)	Medication-adjusted BP traits (SBP, DBP, PP)	Pairwise-independent, LD-filtered (r2 < 0.2) previously reported and novel variants
	B	884	Evangelou2018 (PGS002257)	Medication-adjusted BP traits (SBP, DBP, PP)	Pairwise-independent, LD-filtered (r2 < 0.1) previously reported and novel variants
Dementia	A	57	Najar (Jan 2021) (PGS000812)	Clinically defined Alzheimer’s disease	Variants significant at *p* < 1e^−5^
	B	39	Ebenau (Sep 2021) (PGS001775)	Alzheimer’s disease	Genome-wide significant variants

The Source column lists the paper in which each PRS was derived, along with the PGS ID (Polygenic score ID in PGS Catalog) where available. The “Construction” column outlines the derivation method of each PRS, as described in its source paper. For a brief summary of the dataset(s) where each score was derived, see Supplementary Table 1. For more detailed information about each PRS, please see its source paper.

We examined the overlap in SNPs between PRS-A and PRS-B for each disease, including those in high linkage disequilibrium (LD) (R^2^ > 0.8).

We took care to attempt to avoid the sample overlap issue, a potential pitfall for PRS^24^. Since we planned to calculate PRS in the UKB population (i.e. target cohort), we preferred PRS that were not derived in the UKB population. During the PRS selection stage, we examined the base GWAS cohorts for PRS derivation and attempted to ensure that they do not contain our target cohort (i.e. UKB).

We were able to identify such PRS for breast cancer and dementia, but not PRS-A for SBP in the existing recent literature to the best of our knowledge. We investigated all the available PRS for SBP in the PGS Catalog, and found that UKB was present in all the derivation populations. Given the extensive blood pressure measures and genetic data in the UKB, it is unsurprising that researchers would include UKB in their derivation population for PRS.

### Calculating PRS

We computed the PRS of an individual $j$ by the weighted sum of trait-associated SNPs,

$$PRS_{j} = \mathop \sum \limits_{i}^{N} \beta_{i} *dosage_{ij}$$

where N is the total number of SNPs, $\beta_{i}$ is the effect size (or beta) of SNP $i$, and $dosage_{ij}$ is the number of effect alleles (usually encoded as 0, 1 or 2 in SNP $i$ for individual $j$ for the effect allele).

We applied genetic quality control (QC) pipelines for both SNPs and samples. During SNP QC, we removed ambiguous SNPs (A/T or C/G SNPs with MAF > 0.49) and rare variants with MAF < 0.005; we only retained SNPs with high imputation quality (imputation information score > 0.4) (Supplementary Table 1). During sample QC, we excluded participants who were sex-discordant, outliers for missingness or heterozygosity, or related at 3rd degree or higher, using UKB Data Field 22020.

We then weighted the SNPs that passed our QC using the published effect sizes provided from the source paper for each score, given either in their supplementary materials or made available in the PGS Catalog, to compute PRS for those within the study population for each of the three disease outcomes.

### Quantifying the stability of PRS

In each disease-specific study population, we first calculated the correlation coefficient between each pair of PRS. We then computed the age- and sex-adjusted odds ratios (ORs) at various cut-points (e.g. top 1% or top 5% of the PRS), with the middle quintile of the PRS being the reference group.

We computed two versions of the AUC obtained from two separate logistic regression models for each continuous PRS:

1. Crude-AUC, where only PRS were fitted, adjusting for genetic array, and first 5 principal components (PCs) of ancestry (UK Biobank Field 22009), to reflect the predictive performance from the PRS itself.
2. Multivariable adjusted AUC (Multi-AUC), where the model further adjusted for age and sex (if applicable).

The outcome of these logistic regression models is the disease status (Yes/No), for each of the three diseases. The Crude-AUC measures the predictive ability of the PRS on its own, while the Multi-AUC measures this after having taken account of age and sex.

The continuous net reclassification index (NRI) was used to compare PRS-A with PRS-B in multivariable logistic models (Table 2). The categorical NRI was used in cross-classification of PRS percentile risk categories. Percentage reclassification for participants who experienced the outcome are shown in Supplementary Tables 5–7 and 11–13, for top 1% and top 5% risk categorisations, respectively.

**Table 2:** PRS compared for each outcome and their performance characteristics in the UK Biobank.

Disease (N)	PRS	nSNPs	OR (95% CI)	Crude-AUC (95% CI)	Multi-AUC (95% CI)	NRI (95% CI)	*r*	LD: R^2^ > 0.8 (% overlap)
Breast cancer (171,490)	A	313	3.41 (2.89, 4.03)	0.63 (0.63, 0.64)	0.64 (0.63, 0.64)	0.03 (0.00, 0.05)	0.65	225 (72%)
	B	118,388	3.94 (3.36, 4.61)	0.64 (0.63, 0.64)	0.64 (0.63, 0.65)			
Hypertension (317,581)	A	267	1.83 (1.70, 1.98)	0.57 (0.56, 0.57)	0.69 (0.69, 0.69)	0.16 (0.16, 0.17)	0.66	210 (79%)
	B	884	2.18 (2.02, 2.35)	0.59 (0.59, 0.59)	0.70 (0.70, 0.70)			
Dementia (335,689)	A	57	1.67 (1.30, 2.13)	0.55 (0.54, 0.56)	0.80 (0.79, 0.80)	0.13 (0.10, 0.16)	0.51	13 (23%)
	B	39	2.30 (1.85, 2.87)	0.57 (0.56, 0.57)	0.80 (0.79, 0.81)			

N: number of participants whose PRS score was obtained. nSNPs: number of SNPs in PRS prior to genetic quality control. OR: odds ratio for top 1% versus middle quintile of PRS from multivariable logistic regression model adjusted for age, sex, genotyping array and first 5 PCs. AUC: area under receiver-operating curve. Crude-AUC: only continuous PRS was fitted in a regression model. Multi-AUC: continuous PRS was fitted, further adjusted for age and sex. NRI: continuous net reclassification index obtained from predicted risks by two multivariable logistic regression models that contain age, sex, continuous PRS for this disease, genotyping array and first 5 PCs. The model containing PRS-B is considered the “updated” model. $r$: Pearson correlation coefficient between the two continuous PRS for this disease. LD: number (%) of SNPs in PRS-A which either appear in or are in linkage disequilibrium (R^2^ > 0.8) with SNPs in PRS-B. Breast cancer models are not adjusted for sex because its population is restricted to females. 95% CI for AUC and NRI calculated by bootstrapping.

The 95% confidence interval (CI) of the AUC and NRI were estimated using 1 k bootstrap replications.

### Ethics approval and consent to participate

The UK Biobank study (https://www.ukbiobank.ac.uk) received ethical approval from the North West Multi-center Research Ethics Committee (REC reference: 11/NW/03820). All participants gave written informed consent before enrolment in the study, which was conducted in accordance with the principles of the Declaration of Helsinki. This study has been conducted under the UK Biobank application ID 33952.

### Patient and community involvement

The analyses presented here are based on existing data from the UK Biobank cohort study, and the authors were not involved in participant recruitment. To the best of our knowledge, no patients were explicitly engaged in the design or implementation of the UK Biobank study. No patients were asked to advise on interpretation or writing these results. Results from UK Biobank are routinely disseminated to study participants via the study website and social media outlets.

### Consent for publication

Yes.

### Transparency statement

The lead author affirms that this manuscript is an honest, accurate, and transparent account of the study being reported; that no important aspects of the study have been omitted; and that any discrepancies from the study as planned have been explained.

## Results

Our study populations were N = 171,490 (incident cases = 6347) for breast cancer, N = 317,581 (prevalent cases = 137,649) for hypertension, and N = 335,689 (incident cases = 4460) for dementia. For comparing different PRS, we focused on two aspects: firstly, the consistency of the selected SNPs and performance metrics. Then we assessed the correlation between each pair of PRS, and the extent to which PRS-B gave the same predictions for individuals as PRS-A.

We found that less than 80% of SNPs in PRS-A were represented in PRS-B for all three diseases, after having taken LD into account (R^2^ > 0.8). This is somewhat surprising, as one might expect a newer score (PRS-B) to incorporate most of the previously identified SNPs from PRS-A.

**Figure 1:**
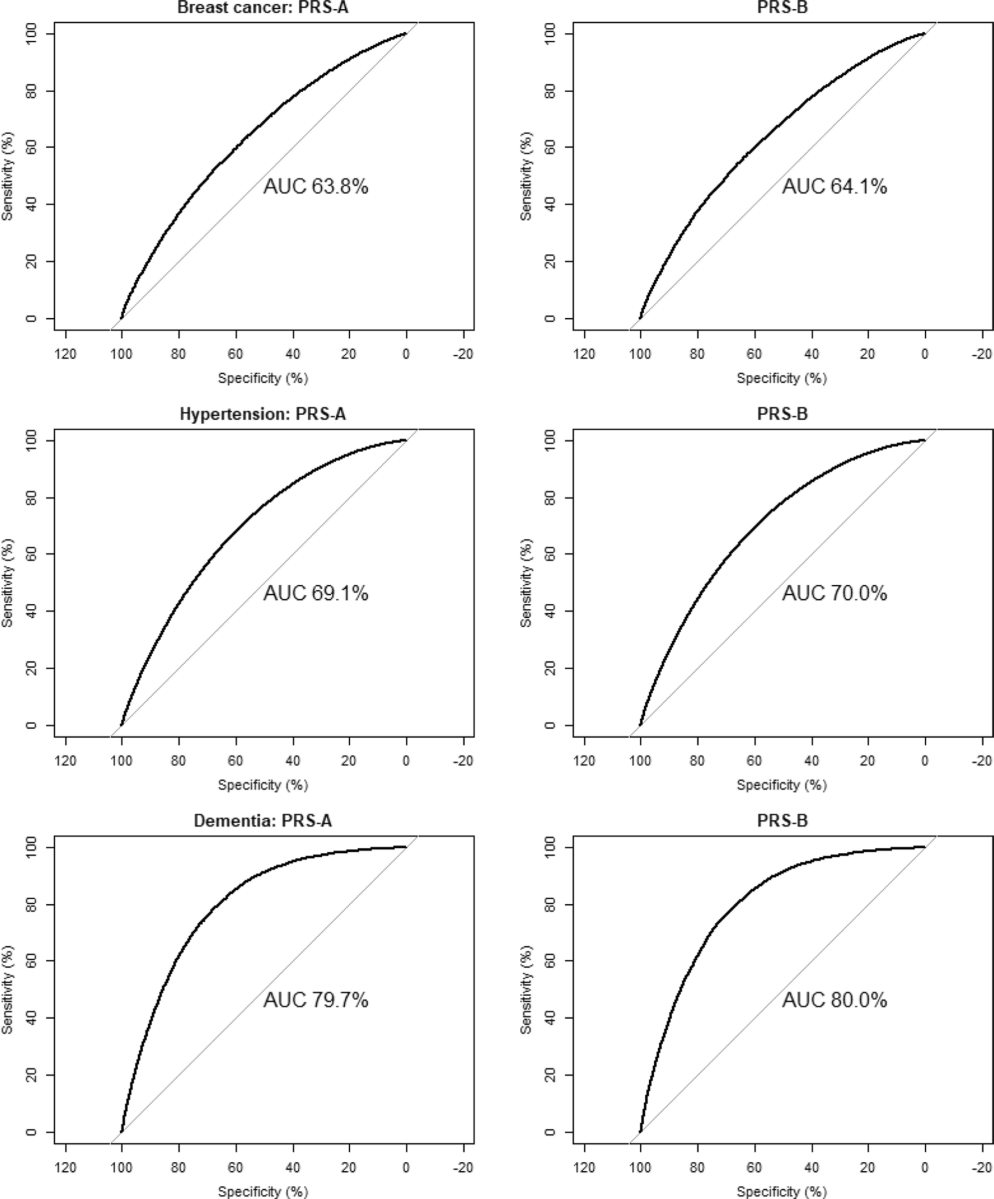
ROC plots obtained from predictions from multivariable logistic regression of age, sex, continuous PRS, genotyping array and first 5 PCs against disease outcome.

Table 2 presents the performance characteristics of each PRS against the corresponding disease outcome in UKB. In each case the more recent PRS-B was associated with a slightly higher OR than the earlier PRS-A. For example, the OR of breast cancer among women in the top 1% compared to those in the middle quintile was 3.41 for PRS-A and 3.94 for PRS-B. Their corresponding AUCs were only minimally different (0.638 vs 0.641), and the ROCs appear almost identical (Fig. 1). Similar results were obtained for hypertension (PRS-A OR = 1.83, AUC = 0.69; PRS-B OR = 2.18, AUC = 0.70) and dementia (PRS-A OR = 1.78, AUC = 0.80; PRS-B OR = 2.30, AUC = 0.80).

The Crude-AUC of PRS were modest, within 0.55–0.64. After including age and sex in the multivariable model, we observed an expected increase in AUC. Despite similar Multi-AUC, PRS-A and PRS-B were not very highly correlated for any outcome, with their Pearson correlation coefficient $r$ only in the range of 0.51 to 0.65. Multi-AUC is noticeably higher than Crude-AUC for dementia, whereas such improvement is less prominent for breast cancer and hypertension. The likely explanation is that age is a more highly influential risk factor for dementia, but less so for breast cancer and hypertension.

Table 2 also shows NRI with PRS-B being the updated model. The positive NRI indicates that PRS-B is better at correctly assigning people to the appropriate risk categories. The small positive NRI is in line with the slight increase in the AUC.

Compatible with these correlation coefficients, there was substantial reclassification of predicted risk according to percentiles of PRS-A and PRS-B for all three diseases. Table 3 shows that for women in the top 1% of breast cancer risk by PRS-A, only 23.1% were in the top 1% risk of PRS-B. The equivalent percentage was 22.9% and 22.7% for hypertension and dementia, respectively (Supplementary Tables 3–4). We focused on the top 1% of risk because of the widely-promulgated concept that these risks approximate those of the risks for monogenic traits^4^.

**Table 3:** Cross-classification of predicted risk of breast cancer among the whole study population, according to the percentiles of each PRS.

Percentiles of PRS-A (%)	Percentiles of PRS-B						
	< 1%	1–20%	20–40%	40–60%	60–80%	80–99%	≥ 99%
< 1	345 (20.1, 20.1, 0.2)	1140 (66.5, 3.5, 0.7)	176 (10.3, 0.5, 0.1)	44 (2.6, 0.1, 0.0)	9 (0.5, 0.0, 0.0)	1 (0.1, 0.0, 0.0)	0 (0.0, 0.0, 0.0)
1–20	1117 (3.4, 65.1, 0.7)	15,409 (47.3, 47.3, 9.0)	8818 (27.1, 25.7, 5.1)	4696 (14.4, 13.7, 2.7)	2052 (6.3, 6.0, 1.2)	490 (1.5, 1.5, 0.3)	1 (0.0, 0.1, 0.0)
20–40	198 (0.6, 11.5, 0.1)	8788 (25.6, 27.0, 5.1)	9989 (29.1, 29.1, 5.8)	8053 (23.5, 23.5, 4.7)	5210 (15.2, 15.2, 3.0)	2051 (6.0, 6.3, 1.2)	9 (0.0, 0.5, 0.0)
40–60	43 (0.1, 2.5, 0.0)	4648 (13.6, 14.3, 2.7)	7998 (23.3, 23.3, 4.7)	8908 (26.0, 26.0, 5.2)	8050 (23.5, 23.5, 4.7)	4607 (13.4, 14.1, 2.7)	44 (0.1, 2.6, 0.0)
60–80	11 (0.0, 0.6, 0.0)	2098 (6.1, 6.4, 1.2)	5296 (15.4, 15.4, 3.1)	7988 (23.3, 23.3, 4.7)	10,047 (29.3, 29.3, 5.9)	8688 (25.3, 26.7, 5.1)	170 (0.5, 9.9, 0.1)
80–99	1 (0.0, 0.1, 0.0)	497 (1.5, 1.5, 0.3)	2006 (6.2, 5.8, 1.2)	4574 (14.0, 13.3, 2.7)	8736 (26.8, 25.5, 5.1)	15,674 (48.1, 48.1, 9.1)	1095 (3.4, 63.8, 0.6)
≥ 99	0 (0.0, 0.0, 0.0)	3 (0.2, 0.0, 0.0)	15 (0.9, 0.0, 0.0)	35 (2.0, 0.1, 0.0)	194 (11.3, 0.6, 0.1)	1072 (62.5, 3.3, 0.6)	396 (23.1, 23.1, 0.2)

Number of participants are shown as n (col%, row%, cell%). Higher percentiles of PRS indicate increased risk of breast cancer; “≥ 99%” percentile corresponds to the top 1% risk.

For participants in the top 5% (a more relaxed risk category than the top 1%) of risk for breast cancer by PRS-A, only 35.7% were in the top 5% risk by PRS-B. The equivalent percentage was 35.8% and 40.0% for hypertension and dementia, respectively (Supplementary Tables 8–10).

## Discussion

The clinical utility of PRS depends on the clinical validity of the predictions. Clinical validity is not only dependent on the information PRS provides on the risk of future events, but also on the stability of these estimates. Here, we demonstrate that for three common conditions (breast cancer, hypertension and dementia), the risk estimates derived from different PRS would result in very different information on risk of future disease being provided to a high proportion of individuals. Choice of the PRS may also influence the use of PRS as covariates or effect modifiers in epidemiologic analyses.

We found that the more recent PRS resulted in minimal increases in AUC compared to older PRS, in line with the small improvement measured by NRI. However, the PRS differed substantially in how they assigned participants into risk categories with a substantial proportion of individuals classified at very high risk by one PRS, not so classified by the other PRS. This suggests a major potential problem for the use of these PRS in clinical practice, given the changes in clinical recommendations associated with labelling a person in the same category of risk as a monogenic disorder. Our results demonstrated large differences across all percentiles of risk; although the clinical consequences at the lower percentiles may not be as extreme as at the higher percentiles, the clinical utility will still be reduced by incorrect classification.

The continual growth of large GWAS studies means researchers are more likely to encounter the issue of inter-cohort sample overlap between derivation and target data sets that may artificially increase the concordance of PRS^25^. This was indeed our experience when selecting appropriate PRS. We were able to confirm that most of our selected PRS do not have the sample overlap issue, but not PRS-A for SBP (detail in “Selection of PRS” section). The reason is that the UKB represents by far the largest single study contributing to PRS for blood pressure, and is unlikely to be excluded from any attempt to derive a clinically-applicable PRS for blood pressure. We anticipate that fellow researchers will encounter the same obstacle, and would like to highlight this challenge to the research community.

The sample overlap between the base GWAS (i.e. derivation cohort for deriving PRS) and UKB (i.e. target cohort for calculating PRS) in our PRS-A for SBP is a limitation of our study. As a result, our results on PRS for SBP should be interpreted with the awareness of potential bias introduced by the sample overlap in PRS-A for SBP.

Current methodological development in the sample overlap issue includes the EraSOR (Erase Sample Overlap and Relatedness) method^25^, which is a potential direction for further research to clarify the roles of sample overlap and different statistical methods in the genesis of PRS discordance.

The portability of PRS depends on the characteristics (such as socio-economic status, age or sex) of the individuals in the base GWAS studies, as well as on the GWAS design, even within a single ancestry group^26^. A difference in study characteristics between the PRS derivation and a target cohort could contribute to the disagreement among different PRS that we have observed in this study. The choice of GWAS sample makes implicit assumptions on sample characteristics that may not hold for the prediction set (i.e. target cohort)^26^. An obvious example is different ethnic backgrounds, but we minimized this by restricting to the White British subset of the UKB. Our study further supports the importance of providing study characteristics along with the PRS.

We note that we have not established the reasons for the extent of misclassification between different PRS. It does not appear to be attributable solely to the number of SNPs included in the PRS. We show this for PRS comprised of over 100 thousand versus several hundred SNPs (breast cancer), for PRS composed of hundreds of SNPs (SBP), and for PRS composed of approximately 50 SNPs (dementia). The surprisingly small number of SNPs held in common by different PRS for the same condition published only a year apart indicates that different analytical methods used to derive the PRS accounts for some of the discrepancies in classification; understanding this phenomenon is clearly important for PRS selection in broad clinical practice. The correlations we observed between PRS for the same condition are in the same range as is seen for variation in risk predictors measured several years apart such as blood pressure and serum cholesterol, far from the “fixed” or “one-time” value at birth that is often assumed for PRS.

This phenomenon has been previously described. For instance, Läll^27^ compared the performance of four PRS in breast cancer prediction, noting that some of the correlations were as low as *r* = 0.3, and observed that a “metaGRS” of the PRS performed better than any of the individual PRS^28^. After completing our analysis and submitting this paper, we found a preprint^29^ with similar findings for two different phenotypes (cardiovascular disease, and educational attainment) in the UKB, providing further generalizability to our results. However, this issue does not seem to be widely appreciated, and most publications comparing a new PRS with previous versions assert the superiority of the new PRS and do not address the issue of misclassification of risk between PRS.

Our observation shows two PRS that only minimally differ in predictive performance on a population level may substantially differ in terms of individual risk classification, even among individuals with the same continental ancestry. This issue requires careful consideration before utilising PRS in real-world settings, because such an arbitrary element in health care is obviously undesirable. An individual’s genetic profile is generally considered fixed at birth, leading to the widely held conviction that genetic susceptibility is an immutable value; however, our findings show that methodologic decisions in the construction of the PRS can lead to meaningful differences in PRS derived from the genome. Although it is reasonable to expect incremental improvements in any risk prediction algorithm over time, these results suggest there is still considerable uncertainty associated with estimates of risk derived from different PRS for the same disease. It will be important to develop guidelines on best practice in constructing PRS to minimize the extent to which people could be given inaccurate or contradictory information over short periods of time.

## Supplementary Information

Supplementary Information.

## Data Availability

Further summary data can be found in the Supplementary Materials; the authors are happy to provide further information upon the request of individual members of the public. Please note that the UK Biobank does not permit researchers to provide the raw data reported in this paper. However, interested readers are able to request the raw data via application directly to the UK Biobank (https://www.ukbiobank.ac.uk). The analytical script in R is available in at https://github.com/2cjenn/AgrPRS.

## References

[1] Torkamani A, Wineinger NE, Topol EJ (2018). The personal and clinical utility of polygenic risk scores. Nat. Rev. Genet..

[2] Yanes T, McInerney-Leo AM, Law MH, Cummings S (2020). The emerging field of polygenic risk scores and perspective for use in clinical care. Hum. Mol. Genet..

[3] Tellier LCAM, Eccles J, Treff NR, Lello L, Fishel S, Hsu S (2021). Embryo screening for polygenic disease risk: Recent advances and ethical considerations. Genes.

[4] Khera AV (2018). Genome-wide polygenic scores for common diseases identify individuals with risk equivalent to monogenic mutations. Nat. Genet..

[5] Kraft P, Hunter DJ (2009). Genetic risk prediction—Are we there yet?. N. Engl. J. Med..

[6] Chatterjee N, Wheeler B, Sampson J, Hartge P, Chanock SJ, Park JH (2013). Projecting the performance of risk prediction based on polygenic analyses of genome-wide association studies. Nat. Genet..

[7] Wand H, Lambert SA, Tamburro C, Iacocca MA (2020). Improving reporting standards for polygenic scores in risk prediction studies. Nature.

[8] Ding Y (2021). Large uncertainty in individual polygenic risk score estimation impacts PRS-based risk stratification. Nat. Genet..

[9] Lewis CM, Vassos E (2020). Polygenic risk scores: From research tools to clinical instruments. Genome Med..

[10] Knowles JW, Ashley EA (2018). Cardiovascular disease: The rise of the genetic risk score. PLoS Med..

[11] Lewis CM, Vassos E (2020). Polygenic risk scores: From research tools to clinical instruments. BioMed Central.

[12] Mavaddat N (2019). Polygenic risk scores for prediction of breast cancer and breast cancer subtypes. Am. J. Hum. Genet..

[13] Fritsche LG (2020). Cancer PRSweb: An online repository with polygenic risk scores for major cancer traits and their evaluation in two independent Biobanks. Am. J. Hum. Genet..

[14] Bycroft C (2018). The UK Biobank resource with deep phenotyping and genomic data. Nature.

[15] Collins R (2012). What makes UK Biobank special?. Lancet.

[16] Lee A (2019). BOADICEA: A comprehensive breast cancer risk prediction model incorporating genetic and nongenetic risk factors. Genet. Med..

[17] Lakeman IMM (2020). Validation of the BOADICEA model and a 313-variant polygenic risk score for breast cancer risk prediction in a Dutch prospective cohort. Genet. Med. 2020 2211.

[18] Michailidou K (2017). Association analysis identifies 65 new breast cancer risk loci. Nature 2017 5517678.

[19] Warren HR (2017). Genome-wide association analysis identifies novel blood pressure loci and offers biological insights into cardiovascular risk. Nat. Genet. 2017 493.

[20] Evangelou E (2018). Genetic analysis of over 1 million people identifies 535 new loci associated with blood pressure traits. Nat. Genet..

[21] Najar J (2021). Polygenic risk scores for Alzheimer’s disease are related to dementia risk in APOE ɛ4 negatives. Alzheimer’s Dement. Diagn. Assess. Dis. Monit..

[22] Ebenau JL (2021). Risk of dementia in APOE ε4 carriers is mitigated by a polygenic risk score. Alzheimer’s Dement. Diagn. Assess. Dis. Monit..

[23] Kunkle BW (2019). Genetic meta-analysis of diagnosed Alzheimer’s disease identifies new risk loci and implicates Aβ, tau, immunity and lipid processing. Nat. Genet. 2019 513.

[24] Wray NR, Yang J, Hayes BJ, Price AL, Goddard ME, Visscher PM (2013). Pitfalls of predicting complex traits from SNPs. Nat. Rev. Genet..

[25] Choi, S. W., Shin, T., Mak, H., Hoggart, C. J. & O’reilly P. F. EraSOR: Erase Sample Overlap in polygenic score analyses. *bioRxiv* 2021.12.10.472164 (2021). 10.1093/gigascience/giad043 PMC10273836 37326441

[26] Mostafavi H, Harpak A, Agarwal I, Conley D, Pritchard JK, Przeworski M (2020). Variable prediction accuracy of polygenic scores within an ancestry group. Elife.

[27] Läll K (2019). Polygenic prediction of breast cancer: Comparison of genetic predictors and implications for risk stratification. BMC Cancer.

[28] Inouye M (2018). Genomic risk prediction of coronary artery disease in 480,000 adults: Implications for primary prevention. J. Am. Coll. Cardiol..

[29] Muslimova, D., Pereira, R. D., von Hinke, S., van Kippersluis, H., Rietveld, C. A. & Meddens, S. F. W. Rank concordance of polygenic indices: Implications for personalised intervention and gene-environment interplay. *bioRxiv* 2022.05.03.490435 (2022).

